# Factors influencing deprescribing in primary care for those towards the end of life: A qualitative interview study with patients and healthcare practitioners

**DOI:** 10.1177/02692163241261202

**Published:** 2024-06-25

**Authors:** Maike S van der Waal, Saskia CCM Teunissen, Allegonda G Uyttewaal, Cathelijne Verboeket-Crul, Hanneke Smits-Pelser, Eric CT Geijteman, Matthew P Grant

**Affiliations:** 1 Center of Expertise in Palliative Care Utrecht, Julius Center for Health Sciences and Primary Care, Department of General Practice, University Medical Center Utrecht, Utrecht, The Netherlands; 2 Academic Hospice Demeter, De Bilt, The Netherlands; 3 Leidsche Rijn Julius Healthcare Center, Parkwijk, Utrecht, The Netherlands; 4 Department of Medical Oncology, Erasmus Medical Center Cancer Institute, Rotterdam, The Netherlands

**Keywords:** Deprescribing, primary care, end-of-life care, interview study

## Abstract

**Background:**

For people with limited lifetime expectancy, the benefit of many medications may be outweighed by their potential harms. Despite the relevance of reducing unnecessary medication use, deprescribing is poorly enacted in primary care practice.

**Aim:**

This study aims to describe factors, as identified by primary care professionals and patients, that influence deprescribing in the last phase of life.

**Design:**

Semi-structured interviews were conducted and analysed using a thematic approach.

**Setting/participants:**

This study was performed in primary care settings, including general practices, hospices and community care teams in The Netherlands. Purposefully identified primary care professionals (general practitioners, pharmacists, nurses) and patients with limited lifetime expectancy due to advanced chronic illness or cancer and their caretakers were interviewed.

**Results:**

Three themes emerged detailing factors influencing deprescribing in the last phase of life in primary care: (1) non-maleficence, the wish to avoid additional psychological or physical distress; (2) reactive care, the lack of priority and awareness of eligible patients; and (3) discontinuity of care within primary care and between primary care and specialty care.

**Conclusions:**

Deprescribing is an incremental process, complicated by the unpredictability of life expectancy and attitudes of patients and health care professionals that associate continued medication use with clinical stability. Opportunities to facilitate the deprescribing process and its acceptance include the routinely systematic identification of patients with limited life expectancy and potentially inappropriate medications, and normalisation of deprescribing as component of regular primary care, occurring for all patients and continuing into end-of-life care.

**What is already known about the topic?**

- Deprescribing refers to the process of systematically discontinuing medications or reducing their dosages. It is part of goal concordant care and reduces polypharmacy while contributing to improving patient outcomes.
- Despite increased attention and various initiatives around deprescribing in recent years, it is inadequately implemented in primary care.
- Most research of deprescribing is focussed on hospital and nursing home settings, views and experiences in primary care are less extensively researched.

**What this paper adds?**

- This article identifies several key factors impacting deprescribing in primary care, notably a wish for health care practitioners and patients to avoid causing distress, a reactive approach to medication management and discontinuity of care intervening with deprescribing.
- The article identifies that deprescribing may be facilitated through proactive discussions around medication use, incorporating existing guidelines into clinical practice and empowering patients to discuss the utility of long-term medications.

**Implications for practice, theory or policy**

- Deprescribing in primary care should ideally be embedded as a continuous routine process for all patients, not only relevant to end of life care.
- A more prominent role of community and practice nurses in deprescribing, in collaboration with GPs and pharmacists, can improve awareness of elderly and chronically ill patients eligible for deprescribing medications.
- Informing and empowering patients to discuss polypharmacy and use of potentially inappropriate medications with their health care provider could stimulate awareness and acceptance of deprescribing.

## Introduction

Patients with palliative conditions are increasingly vulnerable towards the end of life, and the potential harms of medications can start to outweigh clinical value. Increased prevalence of chronic illness with older age and a focus on preventative medicine result in approximately half of all people aged 65 years and over, using five or more medications.^1,2^ Such polypharmacy poses health risks for patients. It can translate into excessive pill burden, decreased quality of life, falls, adverse events and unplanned hospital admissions.^3–8^

The safe reduction of medication in response to the evolving circumstances of the patient is referred to as deprescribing.^9,10^ It is part of goal concordant care aiming to minimise unnecessary medication use, hoping to reduce adverse events, economic costs and burden on patients and carers.^10,11^ Discontinuation of medication should be considered when the primary indication is no longer present, when treatment goals have been adapted, when the time to benefit for medication exceeds life-expectancy or when adverse events start to pose risks or discomfort.^9,11^ Potentially inappropriate medications may include medicines for cholesterol lowering, stomach protection, bone protection, antibiotics/ antimicrobials, antihypertensives and antidiabetics or medications with anticholinergic effects.^12–15^ Studies showed deprescribing can save health care costs and, although there is ambiguity between studies, it is associated with outcomes like improved quality of life and reduced hospitalisations.^16–21^

Palliative and end-of-life care in The Netherlands is predominantly provided in primary care. General Practitioners (GPs), who represent the first point of contact for patients are supported in this task by nurses (specialist and generalist working in GP practices or community setting), pharmacists and social workers. Potentially inappropriate medications are largely managed in primary care during the last phase of life. However, a Dutch retrospective study of primary care drug prescriptions including 7084 palliative patients in the last 3 months of life showed that 45% of people received repeat prescriptions for medications with questionable or no clinical benefit.^22^ Other studies demonstrated that patients used up to ten medications up until the last day of life,^22–24^ and more than a third of patients at home used preventive medications on the day before their death.^24^ Despite its importance, the real-life practice of deprescribing is poorly enacted in primary care.^24–26^

This interview study aims to detail factors that influence deprescribing in the last phase of life, as identified by health care professionals working in primary care, patients and their caregivers.

## Methods

### Study design

This qualitative study employed semi-structured interviews to explore the factors influencing deprescribing in primary care in the last phase of life, informed by a grounded theory approach.^27^ This study was conducted according to the principles of the Declaration of Helsinki and Good Clinical Practice and reviewed by the institutional review board of the UMC Utrecht (21/498), who did not consider this research subject to the Medical Research Involving Human Subjects Act of the Netherlands. Reporting was conducted according to the Consolidated Criteria for reporting qualitative research (COREQ) checklist.^28^

### Setting

This study was performed in primary care settings in the region of Utrecht, The Netherlands. Hospices and community palliative care teams are considered a component of primary care, in which the existing primary care team continues to be key care providers. The GPs included in the study are supported by general practice-based nurses and nurses specialised in palliative care from the hospice.

## Participants

Primary care health care practitioners who provided care for patients with oncological or chronic illness were eligible for inclusion in this study. For patients, inclusion criteria were advanced chronic illness or cancer, having experience with polypharmacy and limited lifetime expectancy.^29^ Their adult family caregivers were also eligible for the study.

## Sample

Key stakeholders within local primary care networks were purposefully identified. Health care practitioners deliberately identified eligible patients.

## Recruitment

Health care practitioners were invited by e-mail to participate in an interview. All invitees consented. After verbal consent to their health care practitioner, patients or caregivers were approached by the researcher, face-to-face, by phone or by e-mail, to participate in an interview. All invitees consented.

All participants received an information letter and informed consent form and were asked to review and sign the form prior to the interview. For primary care professionals, if written informed consent was not obtained in advance, verbal informed consent was provided prior to the interview.

### Data collection

A team of qualitative researchers, GPs and nurse specialists developed a semi-structured interview guide (with separate guide for patient/carers and health care practitioners; Appendix 1), informed by literature review of deprescribing and following the steps for development of an interview guide described by Kallio et al.^30^ The interview guide comprised of four major lines of inquiry: needs and limitations, practical process, identification of patients and timing and roles and responsibilities. Interviews were conducted face-to-face with participants by MW, except one conducted by CV-C. At the time of the study the female interviewer was a medical student holding a PhD, experienced in research and supervised for performing qualitative studies by MG. Participants were unfamiliar with the interviewer. The interviews were conducted between June and November 2022, audio recorded with field notes made during the interviews and manually transcribed verbatim. Interviews lasted between 20 and 90 min. No repeat interviews were done. Transcripts were not shared with participants for comments or corrections, participants did not give feedback on the findings. This work has utilised interview data from health care providers, patients and carers, that includes the names of individuals, that may be potentially identifiable. The data are thus not publicly available. The data may be made available upon reasonable request, dependent on their use, with enquiries directed towards the corresponding author.

### Data analysis

Data analysis employed inductive thematic analysis, as described by Kiger et al.^31^ Data collection occurred as an iterative process alongside data analysis. Inductive coding was initially performed on the interview transcripts utilising NVivo (QSR International, version 12, 2020), and discussed between the researchers to determine final codes. Two researchers (MG, MW) independently coded transcripts, and together identified themes within the data, which were further discussed and refined with the research group (MG, MW, CVC, GU and HSP). Once the coding strategy was finalised, data were organised into inductive themes representative of the participant’s data. Data saturation was defined as the non-emergence of new themes, informed by inductive thematic saturation of Saunders et al.^32^ Saturation was determined according to the four major lines of inquiry in the interview guide, and when the same instances were repeated without new themes emerging, this line of inquiry was ceased for subsequent interviews. Data saturation occurred when all four lines of inquiry were saturated.^27^

## Results

A total of seventeen interviews were completed. Table 1 details participant characteristics.

**Table 1.** Study participant characteristics.

Participant characteristics	Number of participants, *n* (%)
Patients	4 (23.5)
Female/male	1/3
Age range	52–78
Caregiver	1 (5.9)
Female/male	1/0
Health care practitioners	12 (70.6)
Female/male	10/2
Pharmacist	3 (25.0)
General practitioner	4 (33.3)
Practice nurse	3 (25.0)
Nurse specialist	1 (8.3)
Community nurse	1 (8.3)
<10 years work experience	3 (25.0)

**Figure 1.**
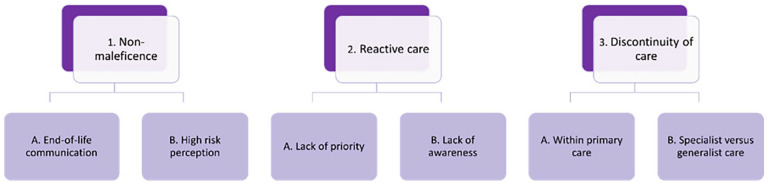
Coding tree.

Three key themes were identified from the interview data are presented in a coding tree (Figure 1) describing pivotal factors that influence deprescribing in the last phase of life in primary care, being: (1) non-maleficence, (2) reactive care and (3) discontinuity of care. How deprescribing was impacted by these themes is summarised below with representative quotes for illustration.

### Theme 1: Non-maleficence

Health care professionals and patients experienced tension between deprescribing and the wish to avoid causing additional emotional or physical distress. Subthemes discussed were end-of-life communication and high-risk perception.

#### End-of-life communication

Health care practitioners were conscious of the precariousness of the last phase of life and wished to avoid additional psychological and existential distress. A critical factor for deprescribing mentioned in all interviews, was a trusted treatment relationship. Patients were open to discuss deprescribing when they felt confidence for the health care provider’s advice to be in their best interest.

*‘The hospice doctor asked what I was using and made a medication list that no longer included them (those medications). Maybe because it isn’t useful. I do trust that doctor to look at these things carefully. That doctor also is the GP of my granddaughter.’ – Patient 1*

Health care practitioners indicated hesitancy discussing deprescribing as they would feel uncomfortable in case predicted life expectancy turned out to be misjudged and feared that this would interfere with the trusted treatment relationship.

#### High risk perception

Many health care practitioners and patients perceived less risk in continuing potentially inappropriate medications than in deprescribing. There was uncertainty due to lack of (awareness) of clear clinical guidelines on deprescribing. Due to the challenges in estimating the survival time of patients, there was fear to deprive, or be deprived, of good care in case someone was to live longer than expected.

Patients and health care practitioners perceived continuing medications was often safer than changing a currently stable situation, even when they were aware of potentially inappropriate medications.

*‘Often it involves vulnerable elderly people with many smaller things, that I think will of course go wrong at some point. But then I don’t know where it goes wrong and then I find it difficult to stop medications, because then you have the illusion, for yourself as well, that you are keeping someone on their feet longer.’ – GP 1*

*‘It makes me feel safe, but I wish I could do without all that mess.’ – Patient 2*

Health care practitioners were cautious to intervene when the patient’s situation was stable, as any events arising after changing medication could be attributed to their intervention. Negative real-life experiences further strengthened this high-risk perception.

*‘Anticoagulants were stopped in the hospice when she developed a thunderous CVA, resulting in hemiparesis and loss of speech. I get the idea of cessation, but it really sucks in case complications arise.’ GP 2*

### Theme 2: Reactive care

Despite participants acknowledging the relevance of deprescribing, cessation of medication was described to be primarily a reactive process.

#### Lack of priority

Deprescribing was indicated to be of lower priority than many other care processes for patients with chronic and incurable diseases. This lack of priority was related to the minimal perceived harms of continuing potentially inappropriate medications, in contrast to more identifiable harms related to stopping medications, especially thrombosis prophylaxis. When patients did develop clinical complaints (i.e. swallowing problems, high blood sugars or hypotension), medication use did gain clinical priority and deprescribing was more likely to occur.

*‘In real-life, what I notice, is that a consultation for a complaint is the trigger for a more careful inspection of medication use. . . .Not that I do not dare to have the conversation, the opportunity is limited by a lack of time and space in the setting that you work in. . . . A medication review can be complex, and the lack of urgency can result in that you let it go.’ – GP 3*

*‘Medication isn’t as important as mental wellbeing. And contact with other people is even more important.’ - Patient 3*

Participants did not perceive that the economic impact of medications was sufficient justification for deprescribing, as costs for continuation of most medications were seen as insignificant.

#### Lack of awareness of eligible patients

Health care practitioners described challenges in identifying patients in their last phase of life for medication review. Medication reviews were often instigated by pharmacists who pre-selected patients to discuss with GPs. Selection criteria were based on polypharmacy and age, with pharmacists lacking insight into patients’ life expectancy.

Practice and community nurses who perform routine disease management for elderly and chronically ill did identify patients with limited life expectancy and managed their medications, however they were not fully aware of the relevance of medication reviews in the context of deprescribing in the last phase of life, and these patients were not routinely communicated to the GP.

Both patients and health care practitioners indicated that raising awareness for potentially inappropriate medication could effectively be initiated by patients themselves. Patients requesting medication reductions were likely to have their medications reviewed and subsequently stopped.

*‘One medicine that I no longer use is morphine. . .stopping was my own decision. The statin was stopped because of side effects. I indicated it was bothering me and then it was stopped. . . .I have seen so many doctors over the years, you do learn from that. I have become more assertive.’ - Patient 4*

*‘I’ve have had a few conversations where people tell me; I don’t need it anymore. I have reached the age of 94. It’s been good, so why am I still taking 15 pills a day? Is this necessary? Then you do get interesting conversations. Indeed, what are we actually doing? Half the tablets you take are to prolong your life, and I can hear you very clearly saying that is something that isn’t a priority anymore.’ – Pharmacist 1*

### Theme 3: Discontinuity of care

End-of-life care, including deprescribing, was identified as primarily the responsibility of GPs in collaboration with other health care practitioners. Discontinuity of care providers, both within primary care and when transitioning between specialist and primary care, limited engaging in deprescribing.

#### Primary care collaboration

Having a deprescribing process in place, with time and staff allocated for medication reviews and follow-up, was thought to greatly facilitate deprescribing. However, none of the health care practitioners identified a work process with time or staff allocated for medication reviews in the context of last phase of life.

*‘I also think the process steps need to be better mapped out. Is the pharmacist involved, the GP, etc. All the steps need to be clear, who does what. Once you discuss with the patient and they agree to start the process, you are now confronted with actually not knowing how to deal with it. Precisely because it is a sensitive issue, continuity of care is important.’ – Practice nurse 1*

If performed, reviews were largely initiated by the GP, while follow-up was allocated to nurses, with limited communication on decisional motives. Monitoring the process of deprescribing was further complicated by many part-time and locum GPs and nurses.

*‘Often when there is an incident, for example a stroke. . . when the patient comes back to us, you see that things have changed. I think that’s quite a pity, there could have been far better cooperation with us. We think in nursing terms. We take a broader view and include the social aspect. The practice nurse could have added value in their care’ – Practice nurse 2*

#### Specialist versus primary care generalist

Primary care health care practitioners described being reluctant to interfere with specialist-initiated pharmacotherapy, feeling less capable of appreciating the risks and benefits of specialised medications.

Hospitalisations during the last phase of life often resulted in additional medications, that were perceived from the primary care perspective to not often align with treatment goals. Although peer consultation was possible, due to time constraints this was not always prioritised. Instead of collaborating with specialists, primary care health care practitioners tended to await the moment patients transitioned back to primary care to consider deprescribing.

*‘We had someone who broke her hip after a myocardial infarction (MI). She returned from hospital with an awful lot of medication, statins, metoprolol, everything. A cardiologist works with cardiology guidelines, if you have a MI; you qualify for the whole nine yards.’ – Nurse specialist 1*

## Discussion

### Main findings

Given the nature of deprescribing at the end of life in primary care, its implementation in practice is invariably complex and affected by multiple systemic, local and personal influences. To improve the deprescribing process and foster acceptance, proactive measures such as systematically identifying patients with limited life expectancy and potentially inappropriate medications are crucial. Normalising deprescribing through positive framing in routine care, starting early and continuing seamlessly into end-of-life stages, creates opportunities for successful implementation. Goals of care discussions should ideally take place alongside the deprescribing process, initiating this process may lead to broader discussions involving advanced care planning. Empowering patients to raise awareness on potentially inappropriate medications is another key step to facilitate deprescribing.

### What this study adds?

In this study patients with limited life expectancy were generally open to deprescribing. A trusted treatment relationship was a precondition for patients to discuss deprescribing. In contrast, health care practitioners seemed more hesitant to disturb the trusted treatment relationship through initiating these discussions. This hesitancy related to uncertainty of life expectancy and high-risk perception of deprescribing. Health care practitioners were described to perceive patients reluctant to engage in discussions on medication use as part of end-of-life care.^33–35^ However, these study results align with findings from patient research showing willingness to discuss medication use in the context of end-of-life care, particularly when supported by their physician.^26,36,37,38,39,40,41^ Strategies targeting and empowering patients to discuss deprescribing, such as sharing information on deprescribing options in advance of a consultation, have been previously shown to be effective^42^ and could be further explored.

The results presented here suggest that the high-risk perception of deprescribing was, at least partially, related to loss aversion; losses were perceived more distressing by patients and health care practitioners than similar sized gains were perceived to satisfy. This phenomenon is linked to ‘pervasive asymmetry’. Deprescribing is likely to suffer from pervasive asymmetry, in which stopping care, even when revealed to be of limited or no benefit, is harder than implementing something new and promising.^43^ It may be beneficial to study the effect of positive framing of deprescribing both in interprofessional and patient communication.

Furthermore, this study shows that identification of patients with a limited life expectancy and use of potentially inappropriate medications can be improved. Currently, initiatives for early identification of patients in their last phase of life have tended to overrepresent people with terminal cancer.^44,45^ The recognition of patients with limited life expectancy and medications could be systematically incorporated into primary care, following the Scottish Key Information Summary (KIS) example, where complex patients are selected to capture clinical (baseline)status and anticipatory care plans in a shared document between various health care practitioners, to better ensure ‘one voice’ in patient and caregiver communication.^46,47^

In addition, in this study, nurses were interested to play a role in deprescribing. Practice and community nurses could play a more prominent role in deprescribing, especially for the elderly and chronically ill. Implementation of deprescribing should focus on existing local and international guidelines on deprescribing, while considering different professional roles in the process.

### Limitations and strengths of the study

This qualitative study was performed in a primary care setting in The Netherlands. It is likely that in different healthcare and cultural contexts issues raised will not be identical, depending on the role of the prescriber, the organisation of the health care system, the expectations of the patient and the treatment relationship.

In this qualitative study we focussed on factors impacting deprescribing in the last phase of life in primary care. The participants represented a broad array of patients and health care practitioners. We approached data analysis from an inductive viewpoint, in which all participant data were analysed together, which identified thematic concordance throughout the data, reaching data saturation using a pre-determined strategy. Whilst the number of participants in this study was not large, given the consistency in themes emerging from the data and saturation, we believe this is justified.^27,32^

This study adds the perspective of multiple primary health care providers and patients, due to its multidisciplinary approach. The qualitative semi-structured approach enabled in-depth discussions of all themes to capture these perceptions. Using an inductive thematic approach, rather than a deductive approach with an existing conceptual framework (i.e. barriers and facilitators) enabled identification of factors that may have numerous influences on deprescribing – such as that continuity of care with one practitioner may limit multidisciplinary involvement and awareness of eligible patients for deprescribing.

## Conclusion

Deprescribing is an incremental de-implementation process. Its complexity is compounded by the unpredictability of life expectancy, and perceptions of patients and health care practitioners that associate continued medication use with clinical stability. Opportunities to facilitate the deprescribing process and its acceptance include the systematic identification of patients with limited life expectancy and potentially inappropriate medications, as well as normalisation of deprescribing using positive framing in a routine manner, starting early, and continuing into end-of-life care.

## Appendix 1

### Interview guide

#### Health care professionals (health care practitioners)

**Table:**

I	Needs and limitations	In case of a patient with limited lifetime expectancy (less than one year) due to terminal illness or very advanced age: - To what extent can you imagine a consultation with you or a colleague to discuss cessation of certain medicines? - What could be reasons to do a medication review? - What factors limit you or your colleague to do such a medication review? - What helps you or your colleagues to have such a conversation? - What do you consider easy or difficult when discussing tapering medication use in this context?
II	Practical process	- How could medication review and subsequent deprescribing fit in the daily care processes? - What steps would you take when considering deprescribing? - What medication would you consider to stop and why? - How do you decide what medication to select for deprescribing?
III	Identification of patients and timing	- Who are eligible for medication review? - When is deprescribing appropriate? Does timing differ for specific medicines? - How do you or would you integrate the context of limited life expectancy? - How can you select patients for medication review?
IV	Roles and responsibilities	- To what extent are you equipped with enough knowledge and skills to perform deprescribing? - Who do you consider most appropriate to perform the process of deprescribing? - Could you indicate the desirable role division for this process? - Do you envision a role for practice nurses, pharmacists, or other care providers?

#### Patients

**Table:**

I	Needs and limitations	Imagine, yourself or a loved one to have limited lifetime expectancy (less than one year) due to terminal illness or very advanced age: - Would you consider when your medication is then reviewed by an health care practitioner? - To what extent can you imagine a consultation with an health care practitioner to discuss cessation of certain medicines, for example when the risk of harm outweigh the benefits? - What factors limit you to have such a conversation - What helps you to have such a conversation? - What do you consider easy or difficult when discussing tapering medication use in this context?
II	Practical process	- How would you like consultations about medication use to take place? - Where would you want these conversations about medication use to take place (at home, at the general practice, etc.)?
III	Identification of patients and timing	- When do you consider it to be appropriate to discuss medication use? - To what extent do you have an idea which medicines could be appropriate to stop and why?
IV	Roles and responsibilities	- Who would you prefer to discuss medication use with? Consider for example your GP, practice nurse, pharmacist, medical specialist, etc. - Who would you not like to discuss medication use with?
